# Ultrasound-guided vs. Non-ultrasound-guided femoral artery puncture techniques: a comprehensive systematic review and meta-analysis

**DOI:** 10.1186/s13089-025-00422-8

**Published:** 2025-03-06

**Authors:** Yi-Chen Huang, Yueh-Hsun Lu, Wei-Yi Ting

**Affiliations:** 1 Department of Radiology, Shuang-Ho Hospital, Taipei Medical University, No.291, Zhongzheng RdZhonghe District, New Taipei City, 23561 Taiwan, R.O.C.; 2 Department of Radiology, School of Medicine, College of Medicine, Taipei Medical University, Taipei City, Taiwan, R.O.C.; 3 Taipei Neuroscience Institute, Taipei Medical University, Taipei City, Taiwan, R.O.C.; 4 Department of Medical Imaging, Fu-Jen Catholic University Hospital, New Taipei City, Taiwan, R.O.C.

**Keywords:** Femoral artery puncture, Meta-analysis, Ultrasound guidance, Body landmarks

## Abstract

**Purpose:**

To compare the effectiveness of ultrasound-guided (US) versus non-US femoral artery puncture (FAP) methods, including fluoroscopy-guided (FL) and non-guided (NG) techniques.

**Materials:**

This meta-analysis included 11 randomized controlled trials and 1 non-randomized retrospective study, comprising a total of 12 studies involving 5534 patients across diverse clinical settings. Studies varied in operator experience, institutional settings, and procedural protocols. Key outcomes assessed included complication rates, vessel access time, first-pass success rates, number of attempts, and the risk of accidental venipuncture.

**Results:**

Analysis of the heterogeneous dataset showed that guided techniques were associated with reduced complication rates compared to NG methods (pooled odds ratio (OR): 0.45, 95% Confidence Interval (CI) 0.28–0.73). US guidance was associated with decreased vessel access time (mean difference: − 16.30 s, 95% CI − 29.83 to − 2.76), higher first-pass success rates (pooled OR: 3.54, 95% CI 2.36 to 5.30), and required fewer attempts compared to non-US techniques. US guidance also showed lower risk of inadvertent venipuncture (pooled OR: 0.22, 95% CI 0.14 to 0.34).

**Conclusion:**

This meta-analysis suggests potential benefits of US femoral artery puncture techniques over non-US methods, while acknowledging significant heterogeneity across studies. The observed advantages in procedural outcomes varied across different clinical settings and operator experience levels. These findings provide setting for institutional decision-making regarding the implementation of guided puncture methods, considering factors such as operator expertise, resource availability, and specific patient populations.

## Introduction

Femoral artery puncture (FAP) is a critical procedure during vascular catheter placement, arterial access, and various invasive diagnostic or therapeutic interventions [1, 2]. It involves inserting a needle through the skin into the femoral artery, typically guided by palpation and anatomical landmarks [2]. This classic technique may cause complications, including hematoma and pseudoaneurysm formation, which can prolong hospital stays, increase healthcare costs, and lead to patient dissatisfaction.

Femoral puncture often relies on palpation and localization using body landmarks, including bony landmarks and skinfolds [2]. The success and complication rates of palpation-based (PB) and landmark-based (LB) techniques, which are normally known as non-guided (NG) techniques, vary considerably with operator experience and patient characteristics. Less experienced operators, such as young fellows or residents, may encounter a steeper learning curve in achieving successful punctures, while factors such as calcified plaques, weak pulse, and stenosis may further increase procedural difficulty [3, 4]. These challenges have prompted the development and evaluation of guided puncture methods to enhance the accuracy and safety of FAP.

Offering a real-time visualization of the femoral artery and its surrounding structures, ultrasound helps determine the optimal puncture site, angle, and depth of the femoral puncture [5, 6]. While multiple clinical studies have investigated US femoral puncture outcomes, the reported benefits compared to LB techniques vary across different clinical settings. A meta-analysis of four randomized controlled trials (RCTs) revealed that ultrasound guidance (US) resulted in lower overall complication rate, higher first-pass success rates, and shorter vascular access time than traditional LB puncture, though study populations and settings were heterogeneous [7]. Fluoroscopy represents another visualization method, enabling operators to track the needle tip during the procedure and potentially avoid vessel wall perforation [8]. Several clinical studies comparing fluoroscopy-guided (FL) puncture with LB puncture have reported varying degrees of improvement in complication rate and first-pass success rate [9–11].

Despite the potential advantages of US and fluoroscopy guidance (FL) puncture methods, important considerations affect their practical implementation. These include not only the increased cost of specialized equipment but also resource requirements for training, maintenance, and potential impacts on workflow efficiency. The effectiveness of these techniques may vary based on institutional factors such as case volume, operator experience, and available resources. While several systematic reviews and meta-analyses have examined specific aspects of femoral artery puncture techniques, a comprehensive comparison focused specifically on US versus non-US approaches (including both NG and FL techniques) remains limited. Therefore, in the current study, we conducted a systematic review and meta-analysis of both RCTs and non-RCTs to evaluate the comparative effectiveness of US femoral puncture versus non-US approaches, encompassing both NG methods and FL, while considering variations in clinical settings, operator experience, and patient populations.

## Materials and methods

We conducted the analyses and reported the results according to the Preferred Reporting Items for Systematic Reviews and Meta-Analyses (PRISMA).

### Inclusion and exclusion criteria

The current study included RCTs evaluating the efficacy and safety of different guiding methods in femoral artery puncture. The patients of the included trials were subjects who required a femoral artery puncture. We excluded trials that (1) did not clearly describe the inclusion and exclusion criteria of the patients, (2) were obscure in the intervention approaches; (3) were without extractable outcomes, and (4) reported duplicate data.

The guidance methods of the femoral puncture considered in this current study were US, FL, and NG (include PB and LB). Institutional Review Board (IRB) approval was not required for this study.

### Outcomes

The primary outcomes of the current study were vessel access time, number of attempts, and first-pass success rate. The secondary outcomes were the incidence of complications and venipuncture.

### Search strategy and study selection

We searched relevant publications from PubMed, Embase, Scopus and the Cochrane Library until May 2024 using the following keywords femoral puncture, femoral artery puncture, angiography, vascular access, cannulation, ultrasound, sonography, US, and sonoguide. The search strategy was ((femoral puncture[Title/Abstract] OR femoral artery puncture[Title/Abstract] OR femoral artery[MeSH Terms]) AND (angiography[MeSH Terms] OR angiography[Title/Abstract] OR vascular access[Title/Abstract] OR cannulation[Title/Abstract] OR ultrasound[Title/Abstract] OR sonography[Title/Abstract] OR ultrasound[MeSH Terms] OR sonoguide[Title/Abstract] OR US[Title/Abstract]) AND (meta-analysis[Publication Type] OR systematic review[Title/Abstract])). No language or country limitations were applied. The “related articles” option in PubMed was used to broaden the search scope, and we meticulously reviewed all retrieved abstracts, studies, and citations. We also recognized additional trials from the references list of relevant papers. All RCTs in the search results were carefully screened. PROSPERO, an online international prospective register of systematic reviews curated by the National Health Service, United Kingdom, has accepted our review protocol (CRD42024350996).

### Data extraction and methodological quality appraisal

Citations from PubMed and the Cochrane Library were managed using Endnote X8 (Clarivate Analytics), with screening conducted based on title and abstract, and full-text articles were obtained for further evaluation. Two reviewers (Wei-Yi Ting, Yi-Chen Huang) independently extracted the reference information (authors and publication year), sample size, intervention methods (US, FL, NG), and outcome data from the included trials. Disagreements were resolved by a third reviewer (Yueh-Hsun Lu). The same reviewers evaluated the methodological quality of each RCT according to the revised Cochrane risk of bias (RoB Version 2.0, released on August 22, 2019) for randomized trials (except for Fukuda [20], which is a case–control study). Five domains—randomization process, deviations from intended interventions, missing outcome data, measurement of outcome, and selection of the reported result—were assessed. The disagreement was discussed and consulted with the same third reviewer for the final judgment. We graded the risk for each potential source of bias as low, some concerns, and high. The overall risk of each trial was then the most severe risk in any of the domains assessed. We graded the overall risk as high risk when three some concerns appeared in the five domains assessed. The quality of Fukuda [20] study was assessed based on Newcastle–Ottawa scale (NOS) for non-randomized studies regarding selection (0–4 points), comparability (0–2 points) and identification of the exposure of study participants (0–3points) independently [12].

### Heterogeneity

We evaluated heterogeneity of the meta-analysis chi^2^ tests using Cochrane Q tests and I^2^ statistics. I^2^ values of less than 25% were considered to indicate mild heterogeneity, those between 25 and 50% indicated moderate heterogeneity and values above 50% were indicative of severe heterogeneity.

### Statistical analysis

Data analyses were performed utilizing RevMan version 5.4 (The Cochrane Collaboration, Oxford, England). We employed a random-effects model to assess significant heterogeneity between studies. We used the odds ratio (OR) to estimate the effect size of categorical outcomes and mean difference (MD) to estimate that of continuous outcomes, both with a 95% confidence interval (95% CI) to quantify the precision and variability.

## Results

### Selection of studies

**Fig. 1:**
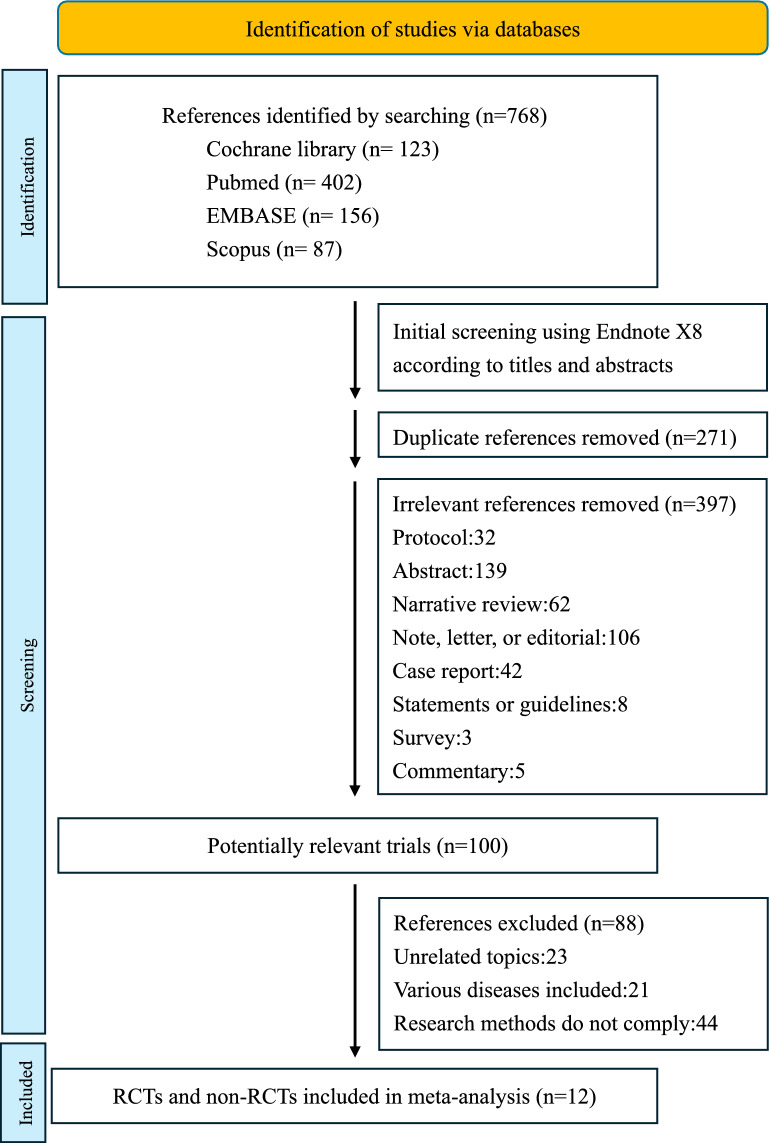
Study flow diagram illustrating the article selection process for the meta-analysis. *RCTs* randomized controlled trials

Our systematic literature search was conducted across multiple comprehensive databases, including Cochrane Library, PubMed, EMBASE, and Scopus, initially identifying a total of 768 references. Following an initial screening process, we removed duplicate references and excluded irrelevant citations based on titles and abstracts. This thorough screening process progressively narrowed our search, ultimately resulting in 100 potentially relevant articles for detailed examination. Following a comprehensive review against our predefined selection criteria, we identified 12 studies that met the inclusion requirements for our meta-analysis (Fig. 1) [4, 9–11, 13–20].

Our meta-analysis included eleven RCTs and one retrospective observational study, encompassing 5534 patients across diverse clinical settings. The included studies varied considerably in their institutional settings, operator experience levels, and specific procedural protocols. A detailed visualization of the selection process is presented in Fig. 1, and the characteristics of included studies are summarized in Table 1, highlighting the variability in study design and implementation.

**Table 1:** Characteristics of the twelve included studies

Author	Year	Organization	Study design	Intervention		Sample Size	Outcome (experimental vs. control)				
				Experimental group	Controlled group		Vessel access time (sec)^c^	Complication^d^	Median number of attempts	First-pass success rate^d^	Accidental Venipuncture^d^
Gedikoglu et al	2013	University	RCT	US	FL	208	68.6 ± 45.1 vs 94.3 ± 66.4	0/108 vs 4/100	1(1–3) vs 1(1–5)	101/108 vs 78/100	No data
Slattery et al	2015	University	RCT	US	FL	100	9:41 vs 7:46/360 vs 300 (med)	2/53 vs 2/47	No data	No Data	No data
Seto et al	2010	1 private nonprofit, 2 university, and 1 government	RCT	US	FL	1,004	185 ± 175 vs 213 ± 194 / 136 vs 148 (med)	7/503 vs 17/501	1.3 ± 0.9 vs 3 ± 3.2	415/502 vs 232/500	12/502 vs 79/500
Stone et al	2020	Private nonprofit	RCT	US	FL	635	80 vs 100 (med)	4/319 vs 4/316	1/347 vs 2/340	257/347 vs 141/340	7/347 vs 32/340
Marquis-Gravel et al	2018	University	RCT + Meta	US	NG^a^	129	No data	1/64 vs 5/65	No data	40/64 vs 31/65	9/64 vs 21/65
Dudeck et al	2004	University	RCT	US	NG^a^	112	208 ± 124 vs 197 ± 165	7/56 vs 10/56	1.93 ± 1.26 vs 2.16 ± 1.62	30/56 vs 23/56	2/56 vs 5/56
Fukuda et al	2021	Private nonprofit	Retrospective	US	NG^a^	404	No data	1/168 vs 34/236	No data	No Data	No data
Chinikar et al	2014	University	RCT	FL	LMG	510	No data	6/279 vs 10/231	No data	No Data	No data
Huggins et al	2009	University	RCT	FL	NG^a^	208	Faster^b^	5/98 vs 3/110	Fewer^b^	No Data	No data
Abu-Fadel et al	2009	University	RCT	FL	LMG	972	105.7 ± 130.7 vs 106.5 ± 152.6	10/474 vs 14/498	2.4 ± 2.2 vs 2.3 ± 2.4	No Data	No data
Koshy et al	2018	Private nonprofit	RCT	US + FL + LMG	NG^a^	313	No data	6/228 vs 4/85	No data	No Data	No data
Katırcıbaşı et al	2018	University	RCT	US	NG^a^	939	33.3 ± 28.2 vs 41.3 ± 64.7	8/449 vs 36/490	1.06 ± 0.26 vs 1.32 ± 0.74	396/449 vs 346/490	8/449 vs 26/490

*US* ultrasound-guided, *FL* fluoroscopy-guided, *LMG* body landmarks, *NG* non-guided, *RCT* randomized controlled trial, *Meta* meta-analysis

^a^Traditional guidance by palpation of the arterial pulse and LMG

^b^Clinical characteristics and procedural factors were similar among the two groups with the exception that fewer needle passes were required and access was achieved faster in the fluoroscopy-guided group

^c^Values are mean ± SD, mean with no SD, or median (med) when appropriate

^d^Data reported as n/N

^e^Data reported as median (range) or mean ± SD when appropriate

### Critical appraisal of the included studies

Two authors (Wei-Yi Ting and Yi-Chen Huang) independently conducted a comprehensive risk of bias assessment across the included studies. The methodological quality evaluation utilized two standardized assessment tools: the Cochrane Risk of Bias tool for RCTs and the NOS for non-randomized studies.

**Fig. 2:**
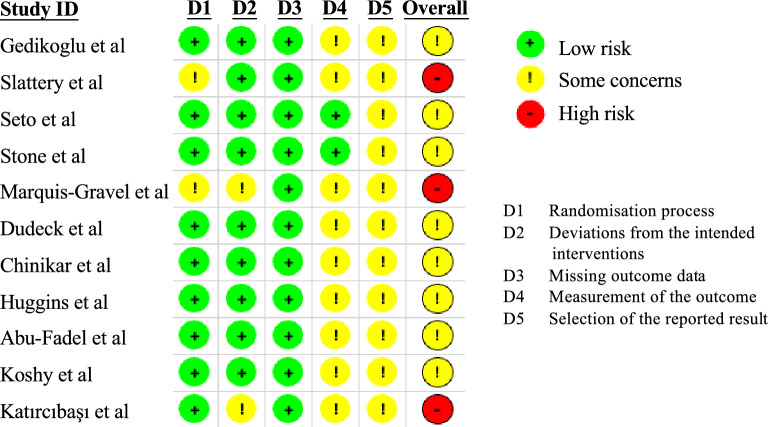
Risk of bias (RoB 2) assessment plot for the included randomized controlled trial studies. The assessment is based on five domains: D1 (randomization process), D2 (deviations from the intended interventions), D3 (missing outcome data), D4 (measurement of the outcome), and D5 (selection of the reported result). Each study is evaluated across these five domains, with a green circle indicating low risk of bias, a yellow circle indicating some concerns, and a red circle indicating a high risk of bias. The overall assessment for each study is provided in the rightmost column

For the RCTs, we systematically evaluated five critical domains, with studies predominantly categorized as having some concerns rather than high risk. No studies were identified with a high blinding risk across the assessed domains. The detailed risk-of-bias assessment for individual RCTs is comprehensively illustrated in Fig. 2. Regarding the non-randomized study by Fukuda et al. [20], the NOS score was 4 out of 9 points, indicating a moderate methodological quality. This comprehensive assessment ensures a thorough evaluation of the included research (Table 2).

**Table 2:** The Newcastle–Ottawa scale scores

Author, year	Study design	NOS score		
		Selection	Comparability	Outcome
Fukuda et al. [21]	Retrospective, single-center observational study	2	1	1

The NOS score is an aggregate score derived from three distinct domains: Selection (with a maximum of 4 points), Comparability (with a maximum of 2 points), and Outcome (with a maximum of 3 points)

*NOS* Newcastle–Ottawa Scale

### Complication rate

**Fig. 3:**
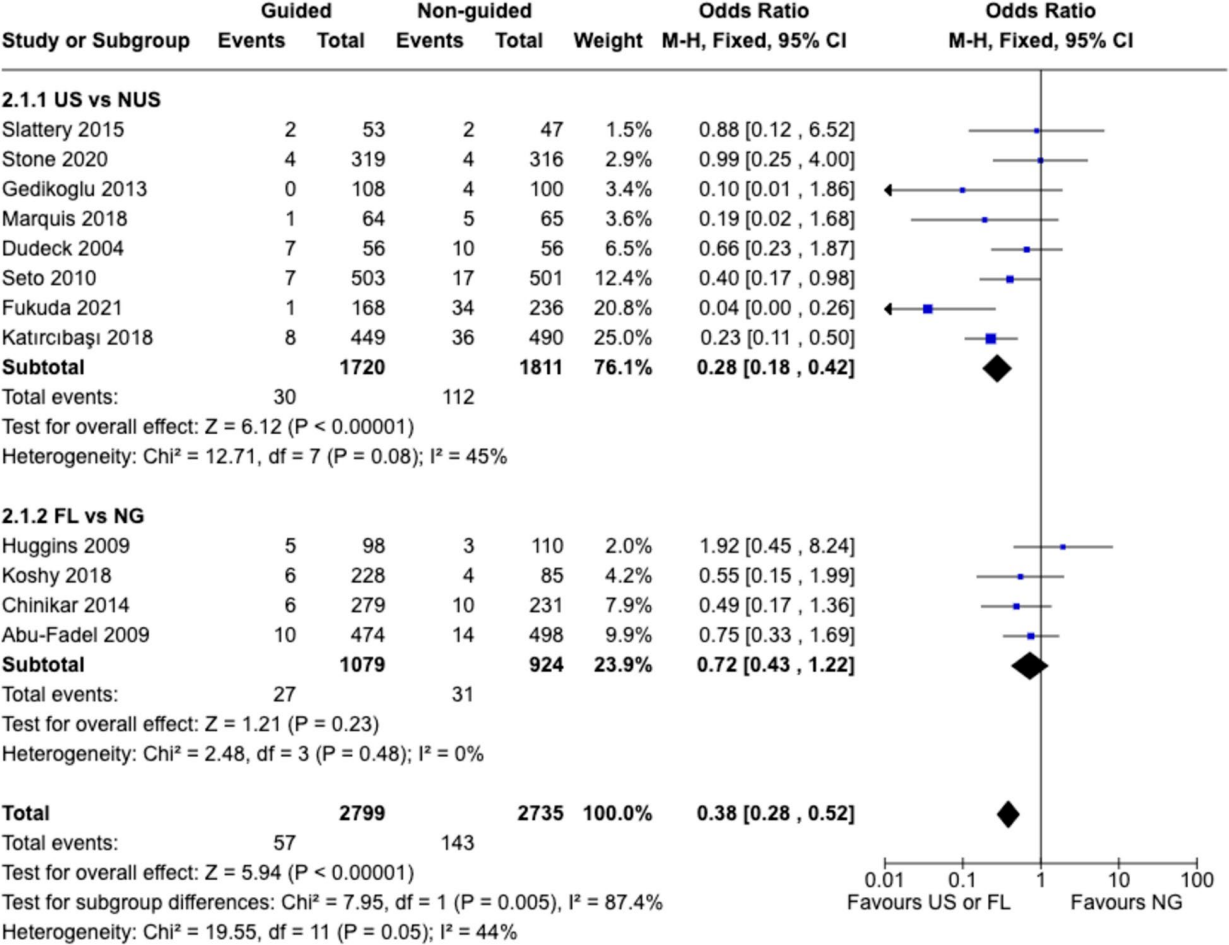
Risk estimates for complication rates. The forest plot presents the ORs and 95% CI for successful femoral artery puncture, comparing US and FL techniques against NG techniques. Each subgroup represents individual studies included in the meta-analysis. The diamonds indicate the pooled ORs and corresponding 95% CIs for both the overall comparison (bottom) and subgroup comparisons (US vs NUS and FL vs NG). An OR below 1 favors the guided technique. The solid vertical line represents the null value (OR = 1). Heterogeneity statistics (Chi^2^, I^2^) and tests for overall effect (Z) are provided to assess the consistency and significance of the findings. *M-H* Mantel-Hoenszel, *CI* confidence interval

Analysis of complication rates revealed varying degrees of association between guided techniques and procedural outcomes. The pooled analysis suggested a trend toward reduced complications with guided techniques compared to non-US methods (pooled odds ratio: 0.38, 95% CI 0.28–0.52, p < 0.00001) (Fig. 3). However, significant heterogeneity was observed among studies, particularly in institutional settings and operator experience levels.

Subgroup analyses revealed differences between guidance methods. The US subgroup showed a trend toward lower complications, with a pooled OR of 0.28 (95% CI 0.18–0.42), with notable heterogeneity among studies (Fig. 3). While some heterogeneity was observed among the US studies, the significant difference between the US and FL subgroups (p = 0.005) highlights the superior performance of US (Fig. 3). The FL subgroup showed less consistent results, with a pooled OR of 0.72 (95% CI 0.43–1.22), with the confidence interval crossed the no-effect line, indicating the possibility of no effect (Fig. 3).

In summary, these findings suggest that the effectiveness of guidance techniques may vary across different clinical settings.

### Vessel access time

**Fig. 4:**
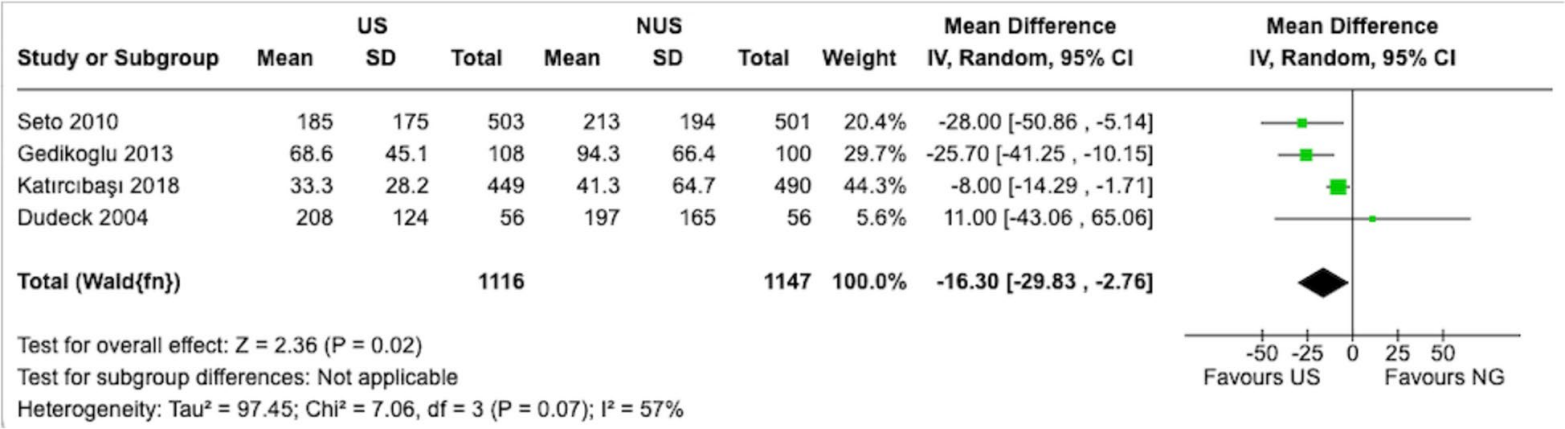
Estimates for time to access the artery. The forest plot displays the mean difference and 95% CIs for vessel access time (in seconds), comparing US and non-US femoral artery puncture techniques. Each subgroup represents individual studies included in the meta-analysis. The diamonds represent the pooled mean difference and 95% CI for the overall comparison. A negative mean difference favors the US technique, indicating a shorter vessel access time. The solid vertical line represents the null value (mean difference = 0). Heterogeneity statistics (Tau^2^, Chi^2^, I^2^) and tests for the overall effect (Z) are provided to assess the consistency and significance of the findings. *SD* standard deviation, *IV* inverse variance method for meta-analysis, *CI* confidence interval

Evaluating the impact of US on vessel access time is critical as quick and efficient vascular access is a key factor in the successful performance of femoral artery punctures. As shown in Fig. 4, analysis of vessel access time from four studies indicated potential differences between US and non-US methods [13, 15, 17, 19]. The mean difference of − 16.30 s (95% CI − 29.83 to − 2.76) indicates that US can substantially accelerate the process of locating and accessing the target vessel, which may potentially improve procedural efficiency and reduce patient discomfort (Fig. 4). These findings highlight the importance of US in enhancing the technical aspects of femoral artery puncture. However, these studies showed considerable heterogeneity in how procedure time was measured, particularly regarding US equipment setup time and operator experience levels.

### First-pass success rate

**Fig. 5:**
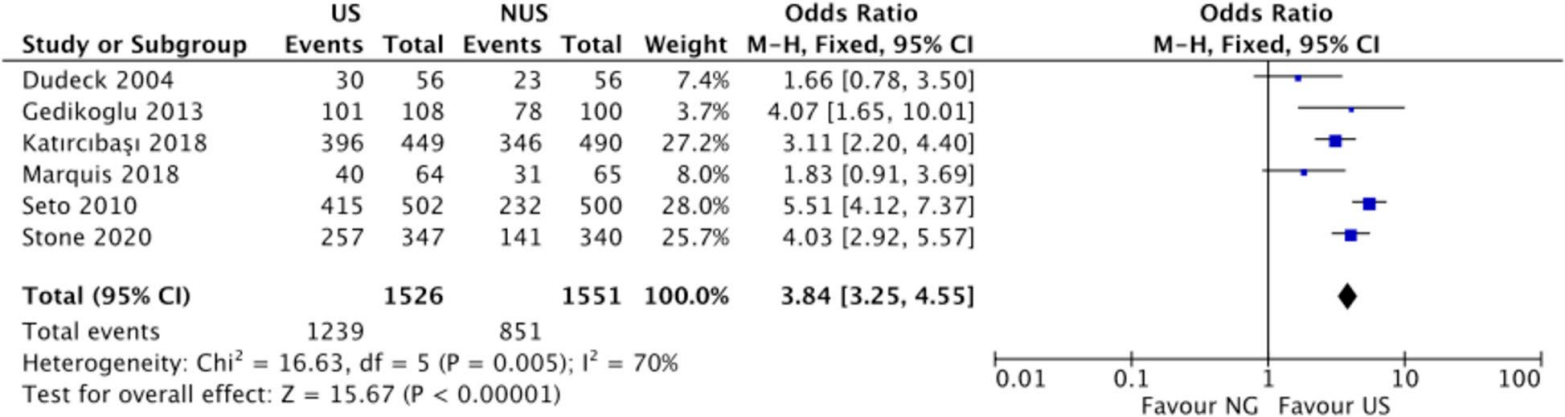
Estimates for success rate at the first attempts. The forest plot displays the ORs and 95% CIs for the first-pass success rates of US femoral artery puncture techniques compared to non-US techniques from individual studies and the pooled estimate. Each study-specific OR is depicted by squares, with the size proportional to the study's weight in the meta-analysis. The horizontal lines represent the 95% CIs for each study, and the diamond represents the pooled OR and its 95% CI. An OR greater than 1 favors the US technique for achieving first-pass success. Heterogeneity statistics (Chi^2^, I^2^) and tests for the overall effect (Z) are provided to assess the consistency and significance of the findings. *M-H* Mantel-Hoenszel, *CI* confidence interval, *df* degrees of freedom

Evaluating the first-pass success rate is a crucial outcome measure as it directly assesses the effectiveness and reliability of US versus non-US femoral artery puncture techniques. As shown in Fig. 5, the pooled analysis of six studies demonstrates a statistically significant difference in first-pass success rates between US and non-US techniques [4, 13, 15–17, 19]. The pooled odds ratio of 3.84 (95% CI 3.25 to 4.55) indicates that the probability of achieving first-pass success is approximately 3.54 times higher with US methods compared to non-US approaches (Fig. 5). Substantial heterogeneity was observed (Chi^2^ = 16.63, p = 0.005, I^2^ = 70%), reflecting differences in operator experience, institutional protocols, and patient characteristics across studies (Fig. 5).

### Number of attempts

Evaluating the number of attempts required to successfully access the femoral artery is a relevant outcome measure as it provides insight into the technical efficiency and precision of US versus non-US puncture techniques. Minimizing the number of attempts is desirable, as it can help reduce patient discomfort, procedural time, and the risk of complications associated with multiple needle insertions.

**Fig. 6:**
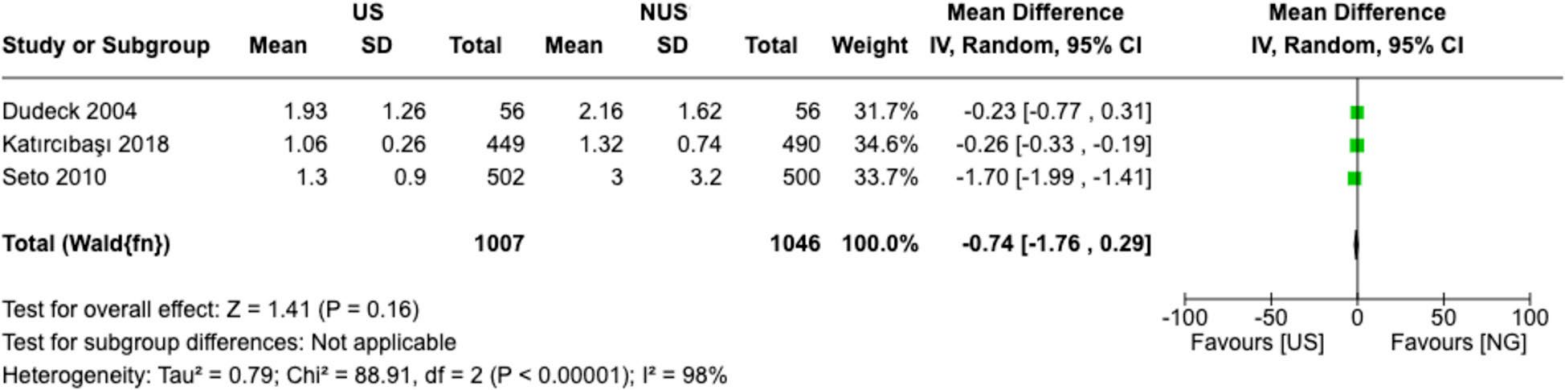
Estimates for number of attempts. The forest plot illustrates the meta-analysis of the number of attempts for femoral artery puncture, comparing US and non-US techniques. Each study-specific mean difference and its 95% CIs are represented by squares and horizontal lines, respectively. The diamond represents the pooled mean difference and its 95% CI. The study weights, based on the inverse variance method, are indicated by the size of the squares. Heterogeneity statistics (Chi^2^, I^2^) and tests for the overall effect (Z) are provided to assess the consistency and significance of the findings. *SD* standard deviation, *IV* inverse variance method for meta-analysis, *CI* confidence interval, *df* degrees of freedom

As shown in Fig. 6, the pooled analysis of three studies demonstrates a trend towards a lower number of attempts with US methods compared to non-US techniques, with a mean difference of − 0.74 (95% CI − 1.76 to 0.29) [15, 17, 19]. While the confidence interval includes the possibility of no difference, the analysis suggests a trend toward fewer attempts with US methods (Fig. 6). This finding is clinically relevant and warrants further investigation with larger sample sizes to better elucidate the potential advantages of US in reducing the number of attempts required for successful vascular access.

### Risk of venipuncture

**Fig. 7:**
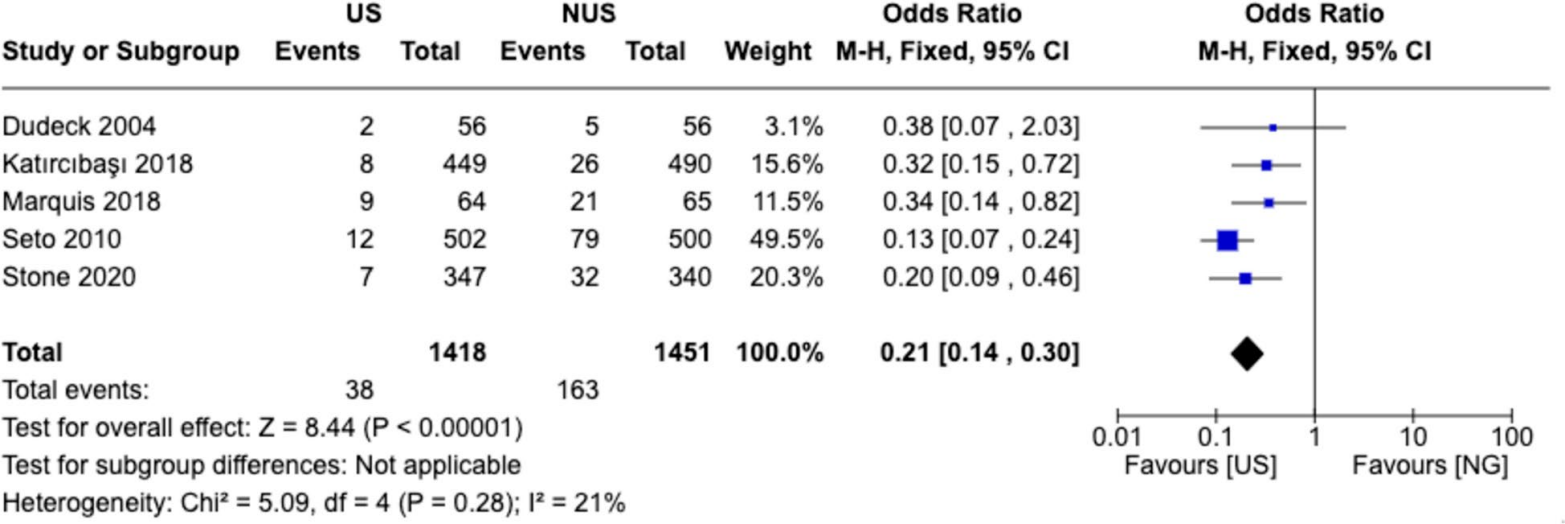
Risk estimates for venipuncture. The forest plot presents the meta-analysis of the risk of venipuncture during femoral artery puncture, comparing US and non-US techniques. Each square represents the OR of individual studies, with the size proportional to the study's weight in the meta-analysis. The horizontal lines represent the 95% CIs. The diamond indicates the pooled OR and its 95% CI. The solid vertical line at 1 represents no difference between the groups, with values to the left favoring the US group (lower risk) and values to the right favoring the NG group (higher risk). Heterogeneity statistics (Chi^2^, I^2^) and tests for the overall effect (Z) are provided to assess the consistency and significance of the findings. *M-H* Mantel-Hoenszel, *CI* confidence interval, *df* degrees of freedom

Expanding on the insights from Fig. 6, we further explore the risk of accidental venipuncture associated with US versus non-US femoral artery puncture techniques as unintended puncture of an adjacent vein can lead to complications such as hematoma and infection. The pooled analysis of data from five studies, involving a total of 2,869 patients, suggested potential differences between US and non-US techniques [4, 15–17, 19]. The pooled odds ratio of 0.21 (95% CI 0.14 to 0.30) indicates that the likelihood of venipuncture is approximately 80% lower in the US group compared to the non-US group (Fig. 7). This finding is statistically significant (Z = 8.44, p < 0.00001), highlighting the substantial safety benefits of utilizing US during femoral artery puncture procedures (Fig. 7).

The individual study results consistently favored the US group, with odds ratios ranging from 0.13 (95% CI 0.07 to 0.24) in the Seto [15] study to 0.38 (95% CI 0.07 to 2.03) in the Dudeck 2004 study (Fig. 7). While individual study results generally favored US guidance, moderate heterogeneity was observed (Chi^2^ = 5.09, I^2^ = 21%), reflecting variations in institutional practices and operator expertise (Fig. 7).

These findings have important clinical implications, as reducing the risk of venipuncture can help mitigate the incidence of associated complications, improve procedural success rates, and ultimately enhance patient safety and satisfaction. These results favor the adoption of US methods as the preferred approach to improve the safety and success of femoral artery access procedures.

## Discussion

### Main findings

Our study suggests that US methods may offer advantages compared to non-US in femoral artery puncture, though these benefits vary across different clinical situations and operator experience levels. The analysis indicates potential benefits in complication rates, vessel access times, and first-pass success rates, though the magnitude of these advantages may differ across different clinical settings and operator expertise levels.

Accidental venipuncture during femoral artery puncture can result in serious complications such as hematoma, pseudoaneurysm, and nerve injury [21, 22]. While US shows promise in improving puncture accuracy and potentially reducing complications, the degree of benefit may vary based on operator experience and institutional factors. The potential advantages in procedure times, radiation exposure reduction, and patient satisfaction need to be considered within the context of specific clinical settings and operator expertise.

The evidence from prior systematic reviews and meta-analyses, including our study, suggests advantages of US in femoral artery access procedures, though with important caveats. The Femoral Arterial Access with Ultrasound Trial (FAUST) by Seto et al. [15] and the study by Katırcıbaşı et al. [19] demonstrate varying degrees of benefit in vascular complications and first-pass success rates [15, 19]. Similarly, randomized clinical trials by Nguyen et al. [23] and Rashid et al. [24] provide additional context for understanding these benefits in specific clinical scenarios [23, 24].

The implementation of US involves important practical and economic considerations [3, 25, 26]. While ultrasound machines are generally accessible and portable, the total cost of implementation extends beyond equipment acquisition to include training requirements, maintenance, and potential impacts on workflow efficiency [27]. The learning curve for US varies among operators, and institutional factors such as case volume and training programs significantly influence successful implementation. Experienced operators often achieve comparable outcomes with NG methods (PB and LB), as demonstrated by Stone et al. [4]. The choice between ultrasound and NG methods (PB and LB) should consider institutional resources, operator experience, and specific patient populations. The integration of US into training programs may offer particular advantages for beginner operators, potentially leading to improved procedural outcomes when implemented within a comprehensive training framework.

## Heterogeneity and limitations of our study

Heterogeneity observed in our analyses might be attributable to the characteristics of the included trials. First, the included trials in our analysis were conducted at various centers spanning private nonprofit, university, and government settings. Such variability implies varying levels of expertise in using device-guided techniques. Therefore, caution is advised when extrapolating the study results to healthcare settings with differing operator expertise levels. Second, besides differences at the institutional level, the included trials also differed in patient demographic features (gender ratio, age), vascular conditions, and physicians’ specialties. Furthermore, several technical aspects require consideration as potential sources of heterogeneity. The included studies varied in their reporting of procedural details, such as needle and sheath sizes, which could influence success rates and complications. The use of micropuncture needles, which might affect the safety profile of the procedure, was inconsistently reported across studies. Additionally, while US procedures demonstrated superior outcomes, the studies did not uniformly account for ultrasound machine setup time in their procedural duration calculations, potentially affecting the interpretation of time efficiency. The technical approach to ultrasound guidance also varied, with studies not consistently specifying whether in-plane or out-of-plane techniques were employed, each having distinct learning curves and potential advantages in different clinical scenarios.

Our analysis was also limited by insufficient subgroup data for specific patient populations. While body composition and vascular disease severity likely influence procedural success and complication rates, most studies did not stratify outcomes based on these factors. Specifically, the comparative effectiveness of US versus NG approaches in patients with obesity or severe atherosclerosis -populations that might particularly benefit from image guidance—could not be adequately assessed due to limited reporting of these subgroup analyses in the primary studies.

Another significant limitation of our meta-analysis is the lack of data on patient-centered outcomes. While we extensively analyzed technical success rates, procedural times, and complications, the included studies did not consistently report patient-reported outcomes such as procedural pain, anxiety levels, or overall satisfaction with the procedure. The absence of standardized assessment tools for patient experience across studies represents a notable gap in our current understanding of the comparative effectiveness of US versus non-US techniques from the patient’s perspective. Future studies should incorporate validated pain scales and patient satisfaction measures to provide a more comprehensive evaluation of these techniques. Such patient-centered data would be particularly valuable for informed decision-making and could potentially influence the choice of approach in different clinical settings and patient populations.

While the heterogeneity observed in our meta-analysis of ultrasound- and fluoroscopy-guided versus traditional non-guided femoral artery puncture techniques highlights important considerations for generalizability, it is essential to recognize the complexity of clinical scenarios. Our included trials did not compare different vascular conditions, especially atherosclerosis severities, vascular stenosis and BMI (which may affect skin to artery distance). The significant variability in patient demographics and vascular conditions commonly encountered in clinical practice underscores the importance of considering these factors when interpreting our findings. The variability in operator expertise levels across different centers may affect their familiarity with the devices. For example, in specialized healthcare settings where operators undergo standardized training programs for device-guided techniques, the variability in operator expertise levels across different centers may not pose as significant a concern. In such contexts, the findings of our study may hold more consistent implications and applicability. Therefore, while acknowledging the potential limitations of our analysis, it is important to consider these factors when interpreting and applying our results in diverse clinical settings.

## Conclusion

In conclusion, the use of US techniques reduces complication rates compared to non-US methods, and US exhibits superior benefits over non-US in first-pass success rates, vessel access time, and attempts required for successful puncture. Ultrasound guidance significantly lowered the risk of accidental venipuncture, highlighting its significance in minimizing complications during procedures. Future research should explore the clinical implications of guided techniques in femoral artery puncture. We recommend US technique as the primary approach in femoral artery puncture for a better patient care with consistent quality.

## Data Availability

The datasets used and/or analyzed during the current study are available from the corresponding author on reasonable request.

## Abbreviations

FAP: Femoral artery puncture
PB: Palpation-based
LB: Landmark-based
NG: Non-guided
RCTs: Randomized controlled trials
FL: Fluoroscopy-guided
FL: Fluoroscopy guidance
PRISMA: Systematic reviews and meta-analyses
NOS: Newcastle–Ottawa scale
OR: Odds ratio
MD: Mean difference
US: Ultrasound-guided
US: Ultrasound guidance
